# Development of a mobile low-field MRI scanner

**DOI:** 10.1038/s41598-022-09760-2

**Published:** 2022-04-05

**Authors:** Sean C. L. Deoni, Paul Medeiros, Alexandra T. Deoni, Phoebe Burton, Jennifer Beauchemin, Viren D’Sa, Eddy Boskamp, Samantha By, Chris McNulty, William Mileski, Brian E. Welch, Matthew Huentelman

**Affiliations:** 1 Advanced Baby Imaging Lab, Rhode Island Hospital, Warren Alpert Medical School at Brown University, Providence, RI USA; 2 Department of Diagnostic Radiology, Warren Alpert Medical School at Brown University, Providence, RI USA; 3 Department of Pediatrics, Warren Alpert Medical School at Brown University, Providence, RI USA; 4 New England Collision, Seekonk, MA USA; 5 Hyperfine, Guilford, CT USA; 6 Philips North America, Cambridge, MA USA; 7 Neurogenomics Division, Translational Genomics Research Institute, Phoenix, AZ USA

**Keywords:** Neuroscience, Biomedical engineering

## Abstract

Magnetic resonance imaging (MRI) allows important visualization of the brain and central nervous system anatomy and organization. However, unlike electroencephalography (EEG) or functional near infrared spectroscopy, which can be brought to a patient or study participant, MRI remains a hospital or center-based modality. Low magnetic field strength MRI systems, however, offer the potential to extend beyond these traditional hospital and imaging center boundaries. Here we describe the development of a modified cargo van that incorporates a removable low-field permanent magnet MRI system and demonstrate its proof-of-concept. Using phantom scans and in vivo T_2_-weighted neuroimaging data, we show no significant differences with respect to geometric distortion, signal-to-noise ratio, or tissue segmentation outcomes in data acquired in the mobile system compared to a similar static system in a laboratory setting. These encouraging results show, for the first time, MRI that can be performed at a participant’s home, community center, school, etc. Breaking traditional barriers of access, this mobile approach may enable imaging of patients and participants who have mobility challenges, live long distances from imaging centers, or are otherwise unable to travel to an imaging center or hospital.

## Introduction

For many clinical neurological applications, magnetic resonance imaging (MRI) is the modality of choice for identifying potential pathology. The continued improvements in MRI technology, which include exquisite spatial resolution, sensitivity to diverse aspects of tissue microstructure, and detailed mapping of tissue metabolites, however, have come at the cost of increasing main magnetic field strengths, improved gradient hardware, advancing radio frequency (RF) coil technology, and the development of accelerated acquisition techniques. Unfortunately, these hardware and technological gains have come at the expense of mobility. To accommodate these advancements, MRI systems have become bigger, heavier, and more power demanding, limiting them to higher income settings such as large urban hospitals or well-equipped research universities. Further still, the proliferation of high field and advanced gradient strength systems has mainly occurred in the ‘global north’, i.e., the higher income countries within North America, the United Kingdom, Europe, China, and Australasia.

In contrast, neuroimaging techniques like electroencephalography (EEG) or functional near infrared spectroscopy (fNIRS), offer the ability to study brain function, electrical activity, and/or cerebral metabolism whilst being portable and having lower cost. The portability and lighter footprint of these modalities further allow them to be used at point-of-care settings, such as in a doctor’s office or, in research settings, within an individual research lab. Unfortunately, while these techniques are undoubtedly valuable in the clinical observation and treatment of epilepsy or other seizure disorders^1,2^, monitoring patients in the intensive care unit^3^, or during sedation^4^, the lack of structural neuroanatomical information is limiting.

Point-of-care MRI is challenged by significant infrastructure requirements. In addition to the initial cost of an MRI scanner itself (commonly ~ $1 M/T), MRI systems require dedicated rooms with electromagnetic shielding and perimeters large enough to avoid interference with individuals with cardiac pacemakers, insulin pumps, prosthetics, or other metallic implants or MRI contraindications. For systems with high gradient performance, significant power delivery and advanced cooling systems are also needed. Whilst many newer MRI systems make use of sealed low-helium magnet designs, the majority of installed systems require liquid helium refills to maintain their low operational temperatures, which require dedicated supply chains with storage, delivery, and handling infrastructure.

Within the context of neuroimaging research, the increasing infrastructural needs of MRI, contrasts with the broader trends in public health research towards lower-cost and accessible data collection using wearable and non-invasive technologies. Large-scale neuroimaging initiatives, such as the UK Biobank, the various Lifestage Connectome projects, the Alzheimer’s Disease Neuroimaging Initiative (ADNI), and the Adolescent Brain and Cognitive Development (ABCD) aim to unlock important new understanding of neurodevelopment and neurodegenerative processes. However, these studies face significant challenges in ensuring diverse and representative study populations. The centralization of high-end imaging systems to major cities and urban settings means that participating individuals and families are often skewed towards higher education and socioeconomic demographics^5–7^, and lack inclusion of rural participants and/or those with mobility and transportation challenges, or families with school, daycare, work, or other time commitments that preclude attendance at lengthy study visits.

Outside of North America, Europe, and other high-income countries (HICs), the limited presence and access to MRI systems in low- and middle-income settings (LMICs) has often precluded its use in global health studies aimed at understanding the impact of poverty, malnutrition, sanitation, and other environmental adversities on child neurodevelopment. For example, while the US has nearly 1 MRI scanner per 25,000 inhabitants, India and other countries in Southeast Asia and Sub-Saharan Africa have less than 1/50th of this number for the same population density^8^. As a result, alternative imaging methods, such as EEG and NIRS have become the de facto standard owing to their lower costs and increased mobility.

While high field strength systems offer a range of imaging methods (e.g., anatomical, diffusion, functional, and spectroscopy) with multiple imaging contrasts available for each (e.g., T_1_, T_2_, or proton density weighted anatomical imaging, T_2_ or T_2_* functional image, etc.), it is important to note that commerically avaialble low-field systems (e.g., Hyperfine) offer a smaller repretoire of options with focus on T_1_ and T_2_ weighted anatomical imaging and diffusion coefficent imaging. Thus, current low field systems are not replacements for high field systems in all neuroimaging studies. However, they may be suitable for studies such as ADNI, which have focused on structural changes, or as complements to HBCD and other studies.

Current ‘mobile’ MRI systems are built around 1.5 T magnets and require 18-wheel haulers that can only travel on high weight-capacity roadways and must be parked on level and reinforced pads. Like their fixed brethren, these systems require specially-installed 480 V 3-phase electric supplies and are limited to area neasr hospitals, out-patient clinics, or other specially designed centers. Low-field MRI systems, such as those that operate with a permanent or resistive magnetic field between 50 and 200 mT, offer the potential for more portable and accessible MRI, due to their lower weight, reduced electrical needs, and permanent magnetic field. By exploting these characteristics, we sought a more flexible approach that would allow “go almost anywhere” scanning and achieve three functional aims: (1) Travel on local and dirt roads without a commercial license to allow access to rural communities; (2) Use portable or fixed power; and (3) Maintain the ability to easily load and unload the scanner for imaging in or outside the vehicle (e.g., in a family garage, in a school, or in an assisted living center). Our approach builds on past prototype work by Nakagomi et al.^9^ who proposed an extremity (elbow) 200 mT imaging device built into a car. Here, for investigational and research purposes, we assess the feasibility of a moderately customized cargo van that incorporates a commercial Hyperfine Swoop 64 mT low-field MRI scanner. The goal of the current work was to demonstrate the feasibility of at-home MRI, and to evaluate the potential for this approach to shift the current center-based approach to MRI towards a more patient/participant-centered design.

## Results

**Figure 1:**
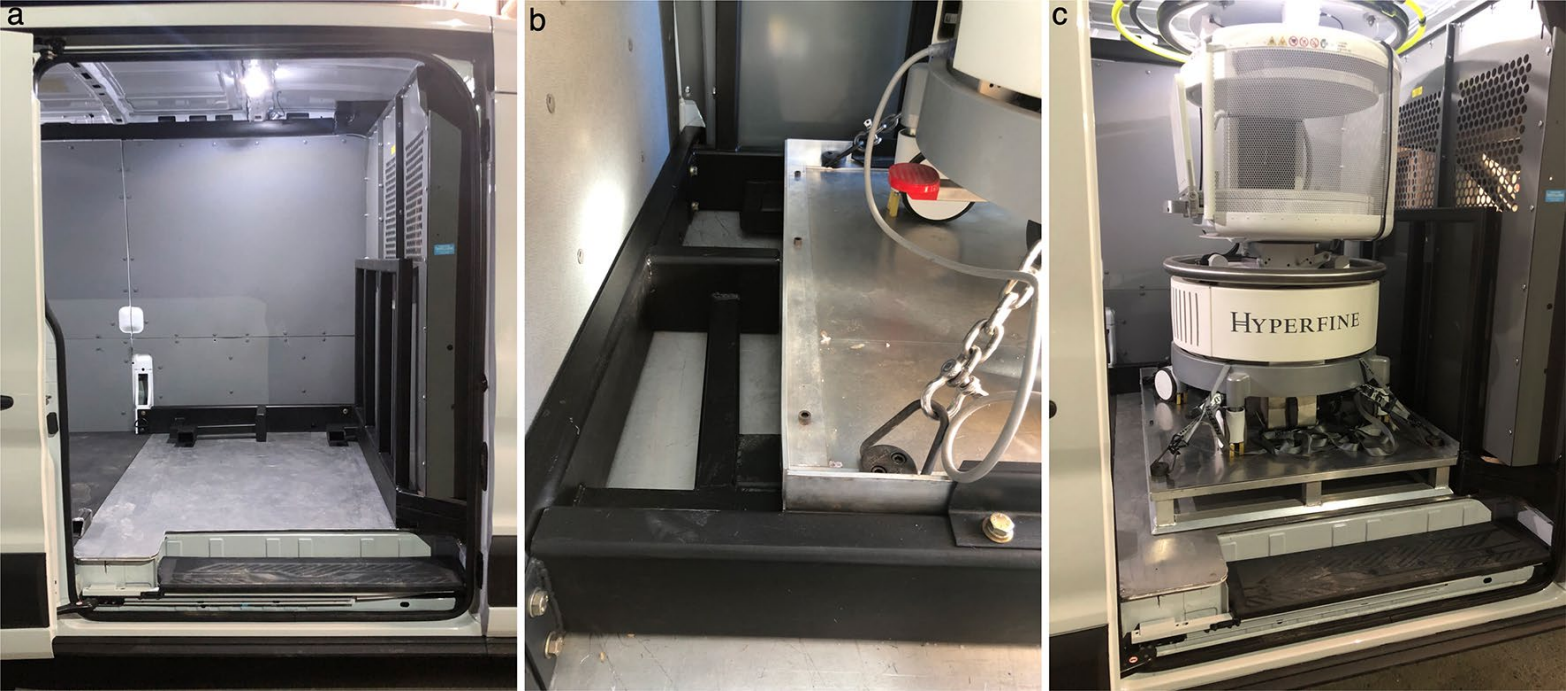
To secure the low-field strength scanner into the van, a reinforced steel and aluminium docking system was developed and welded directly to the frame of the vehicle (**a**) that restrains the device and provides safety to the driver. This system accommodates a custom-designed palette that holds the scanner (**b**), allowing the scanner to moved into and out of the van with a self-loading packer or forklift. To hold the top of the scanner, a halo system was built, minimizing the chance of the scanner tipping and causing vehicle instability (**c**).

**Figure 2:**
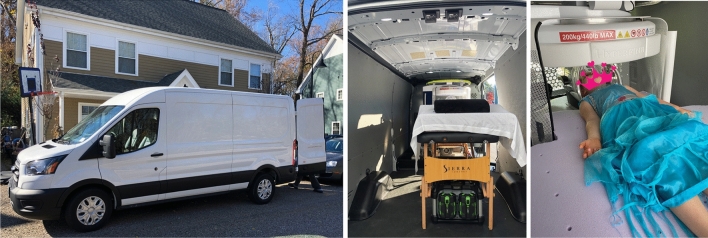
Timeline of scanning from arrival at the participant’s home (left), set up of the scanner bed and powering on of the system using the portable battery supply (middle), and finally scanning of the participant (right). Informed consent and assent was obtained to take, use, and publish these photos of their home and participating child in print and online open-access publications.

**Figure 3:**
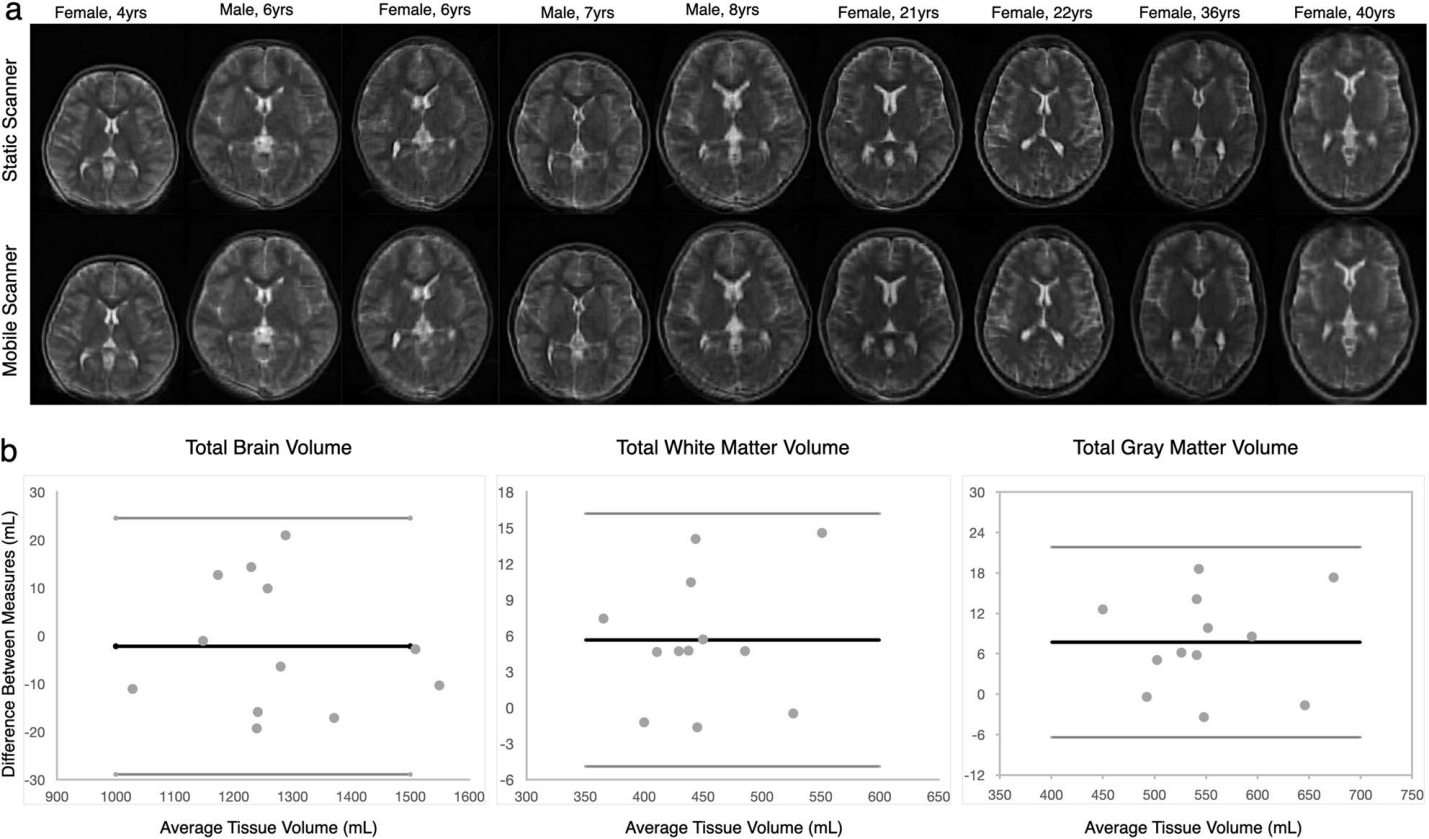
(Top row, **a**) Qualitative visualization of example axial-oriented images of 9 individuals from 4 to 40 years of age scanned in the mobile van and in the static lab-based scanners. There are no visible image artifact differences between the two images. (Bottom row, **b**) Bland–Altman plots for total brain, white matter, and gray matter tissue volume estimates derived following segmentation of the acquired images. No significant bias was observed between the image datasets.

We have built, tested, and demonstrated the first mobile MRI imaging system capable of performing point-of-care neuroimaging. In demonstration of the ability to routinely perform a neuroimaging exam at a participant or patient’s home using a docking scanner configuration (Fig. 1), we show a pictorial timeline of arrival, setup, and scanning at an individual’s residence (Fig. 2), with a comparison of brain images collected of the same individuals in the van and in-lab (Fig. 3). Total time from arrival to scanning is approximately 5 min including attaching to our portable power supply, scanner warm up time, and magnetic field homogeneity checks that are performed as the participant gets ready and is consented for the study.

A video of the first at-home MRI is available at https://www.youtube.com/watch?v=JRfmFpXQnRQ.

**Figure 4:**
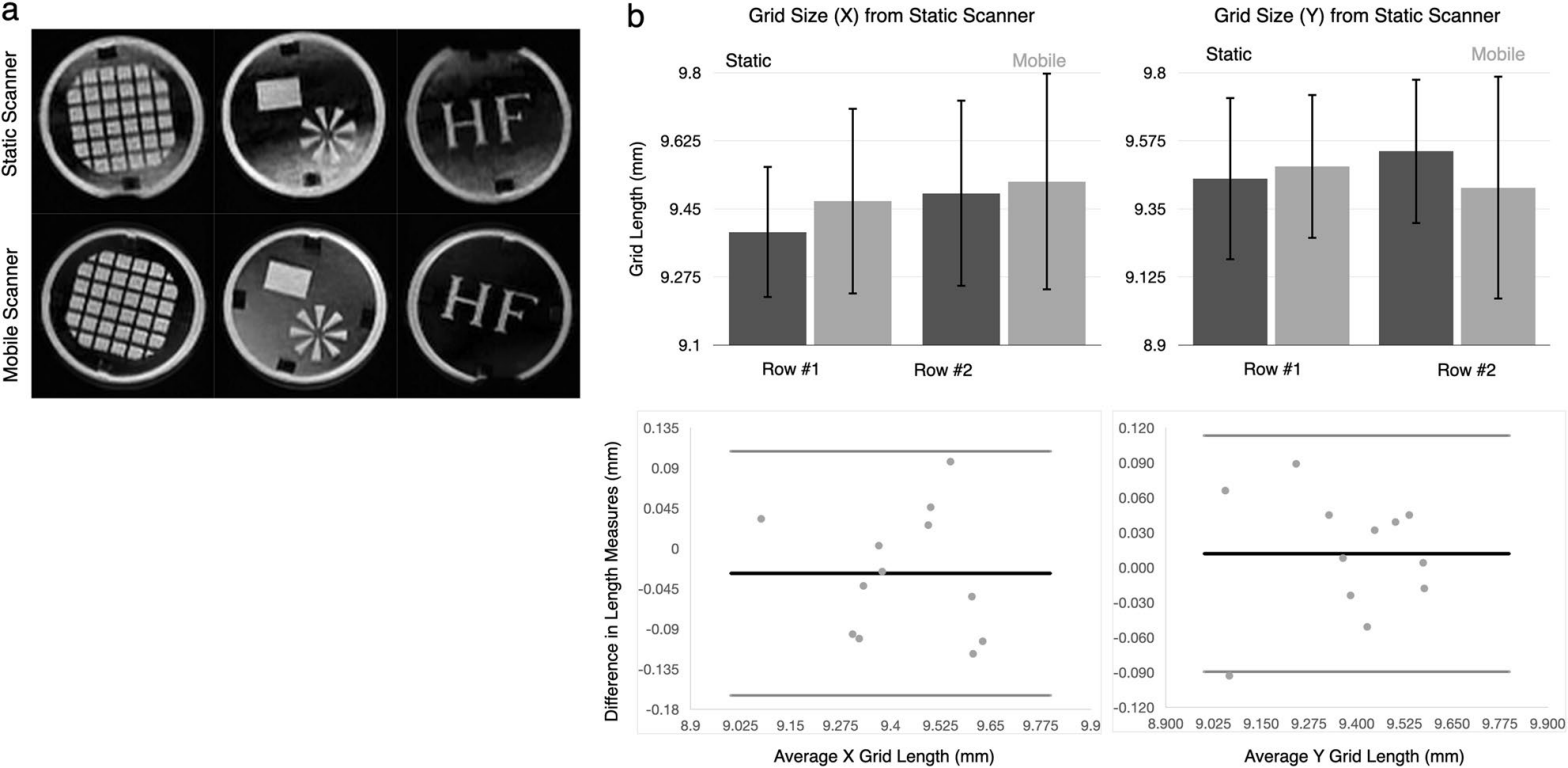
(**a**) Example images of the standard Hyperfine phantom collected in the mobile van and lab-based static scanners. As with the in vivo images, we see no obvious differences in geometric distortion or image quality, which are confirmed in comparisons of the phantom grid size (**b**) along the phantom X and Y coordinates.

In comparison with an in-lab system, we found no significant differences between image segmentation quality (WM: r^2^ = 0.99, *p* = 0.78; GM: r^2^ = 0.99, *p* = 0.77), or phantom image geometric distortion (X Length: r^2^ = 0.84, *p* = 0.68; Y Length: r^2^ = 0.92, *p* = 0.87; Fig. 4). We additionally found no differences in image signal-to-noise (SNR) measures in white matter (163 ± 46 in the static system vs. 177 ± 32 in the van) or thalamalic gray matter (181 ± 35 in the static system vs. 189 ± 40 in the van).

**Figure 5:**
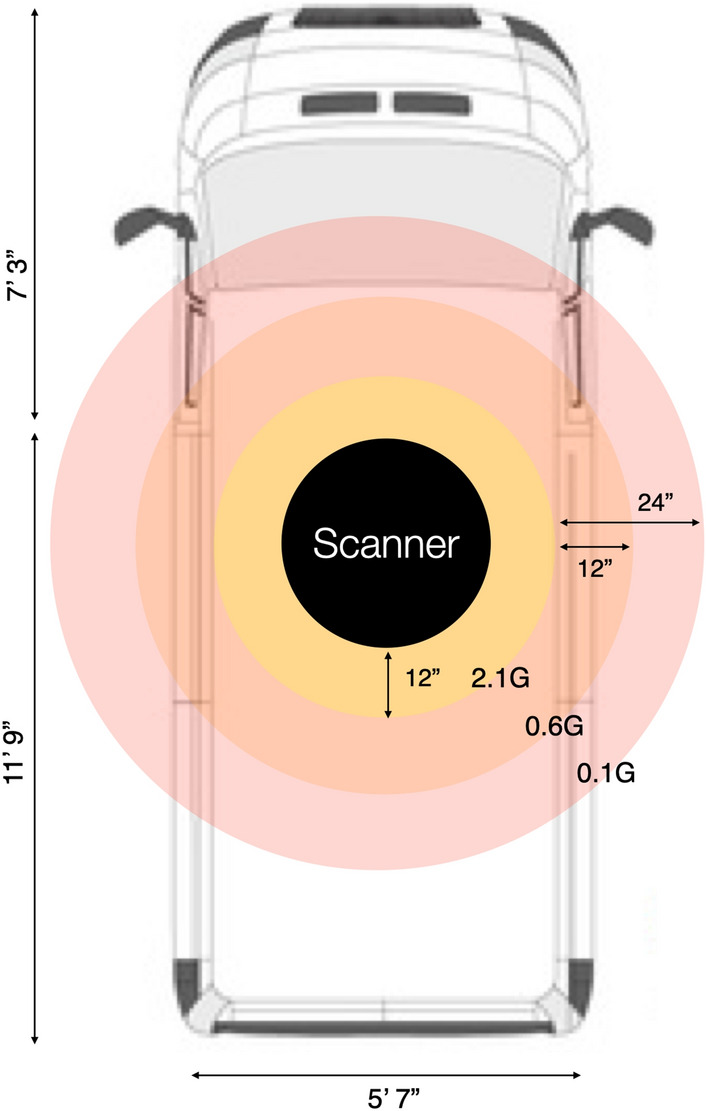
Measured magnetic field around the scanner. We note that at no point outside of the van is the magnetic field greater than 2G, and is near 0 within 2 ft.

Despite the added weight of the MRI scanner and its related accessories, the van is safely below its gross weight rating and is able to travel comfortably at normal road and highway speeds. An additional air-ride suspension is planned to further improve comfort and minimize rocking and shaking of the scanner on rough rural and dirt roads. Measurement of the external magnetic field (Fig. 5) showed it to be below 2 Gauss at all points outside the van (and under 0.6G within 1 foot of the van), removing a potential safety hazard for individuals with pacemakers, implants, or other medical devices sensitive to magnetic fields who might walk by or near the van when parked. Current ICNIRP guidelines place a 5G limit on implemented metal devices and pacemakers (www.icnirp.org).

While mobile labs incorporating EEG and NIRS systems have been used previously for remote neuroimaging^10^ in rural and LMIC settings^11^, MRI has traditionally been too costly, bulky, and complex for mobile imaging applications. Here, however, we show the viability of mobile MRI at relatively low cost. Including the current cost of the Hyperfine system ($50,000), Ford Transit van ($32,000, inc. delivery and licensing), interior modifications ($14,000, inc. pallet, roll-cage, and straps), self-loading lifter/packer to load and remove scanner ($12,000), and associated items (including power generator, battery pack, massage bed, blankets and cushions, $3,500), the total up-font cost of the Scan-a-van is approximately $110,000, which compares favorably to the > $2.5 M cost of a mobile 1.5 T system and trailer. It is anticipated that this price could be further reduced if the scanner could be fixed in the van rather than removable. This would simplify the roll-cage design and eliminate the need for a portable but high weight capacity loader. However, this may also limit the potential applications of the scanner.

## Discussion

We have successfully demonstrated the ability to acquire quality structural neuro MRI data on a low-field MRI system in a mobile platform for the first time. The ability to perform remote neuroimaging at an individual’s or family home, or at a community location (school, assisted living center, library, shopping center, or other) has the potential to substantially increase the number and type of participants enrolled in public health studies that include neuroimaging, as well as in stand-alone neuroimaging-focused clinical studies. Purposefully designed around a commercial van with a large installed base of dealerships (Ford) and capable repair shops with commonly available parts and service items, the platform was designed to be readily serviceable and not require specialized parts or sophisticated knowledge. Further, the system has been designed with accessibility as a primary consideration. The van can travel on almost any road surface (including dirt and gravel within reason), by anyone with a common and non-commercial driver’s license. Similar vans, including the Mercedes Spinter, Hyundai Starex/H-1, Maruti Cargo, and others can be similarly adapted for use in Europe, Southeast Asia, and Sub-Saharan Africa, and is underway.

Operating cost was also a design consideration. Following the initial upfront cost of the van, customization, remote power supply, and scanner purchase (~ $110,000), on-going costs include insurance (fleet insurance, $1200), maintenance ($600), petrol ($1680), and scanner service charges ($35,000 with 3-year research agreement).

Traditionally, large-scale neuroimaging studies such as the Alzheimer’s Disease Neuroimaging Initiative (ADNI)^12^, the Adolescent Brain and Cognitive Development (ABCD) study^13^, and others^14,15^ are comprised of community samples that, although including individuals across dimensions such as socioeconomic status, race, and ethnicity, often self-select only those who are able to travel to the imaging center. This often means that individuals from rural settings, those without reliable and easy access to transportation, or those with time-intensive responsibilities and obligations (e.g., child care or schooling, self-schooling, work, etc.) are unable to participate. Other factors such as the current COVID-19 pandemic have also impacted neuroimaging studies through the closure of many clinical and university research centers and the hesitation of individuals to travel to these centers for fear of becoming infected or sick. It should be noted, howwever, that low-field systems should not be considered as replacedments for high-field systems in all studies. High-field systems offer an array of imaging technqiues and acquisition protocols that are not currently matched on the Hyperfine system. However, in some cases, for example structural neuroimaging and brain volumetric analysis, the Hyperfine system can provide complementary information^16^.

The ability to bring an MRI scanner to a participant, and the ever-increasing ability to perform remote neurocognitive assessments and biospecimen collections, offer the potential to profoundly change how current neuroimaging and neuroscience research is performed, the scope of questions that can be addressed, and the diversity of study populations that can be recruited. By accommodating participant schedules and not requiring them to travel lengthy distances to a study center will allow more traditionally underrepresented individuals and groups to be recruited and retained, helping to address known race, ethnicity, geographic and socioeconomic biases in neuroscience research^17–19^. Moreover, studies focused on specific topics (e.g., agricultural insecticide exposure, drug use and exposures) or study populations (e.g., twins, rare disease, school-age children, elderly individuals with dementia, or individuals with cardiovascular challenges) may benefit from the ability to image participants in rural locations, at daycares, schools, assisted living centers, or in-patient facilities, or without needing to fly them from larger distances to a single imaging center. Although the Hyperfine system is currently capable of four structural image contrasts (T_1_, T_2_, T_2_-FLAIR, and DWI), we believe that as more research groups gain access to these low-field systems we will see steady improvements in image quality, acquisition techniques, and imaging metrics much like we’ve witnessed on high field systems. Similarly, the image quality and spatial resolution of low-field strength images lags behind the best achieveable data from 1.5 and 3 T systems, and remains an area of research interest.

Upcoming studies, such as the HEALthy Brain and Cognitive Development (HBCD) study^20^ and the RECOVER initiative (recovercovid.org) to understand long COVID-19 have an inherent focus on enrolling individuals and families from historically marginalized communities that have suffered disproportionate rates of opioid and other substance use (HBCD) or COVID-19 infections and illness (RECOVER). However, despite this mandate, these studies currently incorporate state-of-the-art high field strength MRI systems and/or other clinical services. Thus, individuals from rural or dis-enfranchised communities face significant hurdles to participation. The ability to bring a portable scanner directly to these individuals represents a paradigm shift in data collection, allowing more diverse and inclusive study populations to be enrolled and followed, as well as expanding access to potential patient populations. Our portable solution, coupled with low-field strength MRI systems, address this access gap. For example, one could envisage a complement to the ADNI study of Alzheimer’s disease in which neuroimaging (and associated neurocognitive assessments) are performed at an assisted living or elderly care facility, enabling participation of individuals without transportation or who may be unable to travel without significant support.

While portable MRI systems based on higher field strength 1.5 T superconducting magnets have been available since the’90s, these systems are designed around 18-wheel haulers that require specially-installed parking pads and electric supplies and, thus, do not afford the accessibility offered by our lower cost and more versatile approach.

Though not investigated or pursued here, there is significant potential for mobile MRI systems in clinical workflows, both for rural participants, or those in areas without easy access to hospital based systems. Examples may include hydrocephalus, in particular shunt revision surgery. Currently, computed tomography, ultrasound, or MRI is used to assess potential blockages near an existing shunt and if revision is needed. A mobile scanner could alleviate congestion on out-patient MRI systems, and reduce the need for patients and their families to travel to an imaging center. A further use could be the clinical monitoring of MS patients, who often require yearly or biannual MRI scans^21^. Again, the ability to bring a scanner to these patients for routine monitoring, or during relapse periods, could ease traffic on clinical scanners while addressing an important need.

Despite this advance, challenges remain. Principal amongst them is the current limitation to structural imaging. Functional, perfusion, and metabolic imaging are important aspects of most neuroimaging studies but are currently difficult or not available on the Hyperfine system. Work towards developing these methods is currently on-going. For functional imaging, further alternatives include incorporation of EEG or NIRS, which can be performed in the low-field system without significant artifacts or image distortion.

## Methods

### Building the Scan-a-van

The aim of this work was to develop an assessable, cost-effective, and safe mobile imaging system capable of reaching most residential locations throughout North America and which could be transferred to LMIC settings in Sub-Saharan Africa and Southeast Asia. As a base, therefore, we chose the Ford Transit High Roof and Extended length 2500 cargo van, which provides ample interior space, a reliable and well-tested EcoBoost V6 engine and 10 speed automatic transmission, and sufficient payload (9500 lbs gross vehicle weight rating) to accommodate the weight of the scanner, participant, and additional equipment. Further, with power steering, brakes and other common features, the van can be driven on local and rural roads (i.e., not restricted to commercial truck routes) without a commercial drivers license (CDL) or any special training.

The Hyperfine Swoop (www.hyperfine.io) MRI system has a permanent main magnetic field of 64 mT, a 5 Gauss boundary diameter of approximately 5 feet, low power requirements, and weighs just over 1400 lbs. The Swoop scanner was developed to increase access to MRI, but is currently only tested and FDA cleared for use at the point-of-care in US medical facilities. While its low weight, small field perimeter, and accessible electric requirements make the system ideal for a mobile application, important safety customizations were necessary to accommodate the system in the van. The system’s weight means it’s capable of causing significant damage or roll-over in the event of a sudden stop or sharp turn. I.e., in a sudden head-on crash, the system would exert a net force 343 N, or approximately 100 times the weight of the 5200 lb van itself. To address this, a reinforced steel roll-cage was designed within the van and welded to the frame in order to keep the scanner stationary and locked in place in the event of a crash. The roll-cage consisted of a bottom steel pallet to hold the scanner and allow loading and unloading from the van using a forklift or loader (Fig. 1); and a docking mechanism to hold the pallet firmly in place and keep it from rolling over in a crash or around corners. A portable and adjustable massage table is used for the patient bed with additional draping and a memory foam mattress to provide comfort and warmth during scanning. The docking device was designed to allow the scanner to be moved in and out of the van for use in schools, community settings, or other communal areas.

To provide power, three options were developed. At a participant’s home, if allowed, power can be drawn from the main electrical supply using an extension cord to the garage or outside 120-V outlet. Where direct access is not permitted or possible, (e.g., at a community center, school, or other public location), an EGO Power + 3000 W portable power station with 4 rechargeable 7.5 Ah batteries provides more than 6 h of continuous scanning and can be loaded into the van without causing artifact or signal disturbance. Finally, a portable propane/gas generator, such as the Champion 3500 W Dual Fuel generator, can be carried along with the scanner to provide additional backup power where needed. In general, we have found the portable EGO power station to be the easiest and most convenient solution.

To allow the scanner to be used into the fall and winter months and avoid participant discomfort or challenges with the scanners recommended operating temperatures (5–30 °C), a heating system was built into the van that could be complemented with a portable electric heater (also run from the portable battery or generator). In the summer months, operating with the rear doors open and an oscillating fan provide sufficient comfort for the short scan duration without impacting scan quality. In cases of extreme heat, a roof-mounted air-conditioning until may be optionally installed. The recommended operating temperature of the system is 15–30 °C, though we have successfuly scanned at − 5 °C and 45 °C without issue.

Our final design consideration was to allow remote loading and removal of the scanner from the van. This was desired for cases where scanning may be performed inside a participant’s home or garage, or in a school, community or assisted living center. This is additionally helpful for participants who have mobility challenges that limit them from being able to climb into the van. The bottom steel pallet was therefore designed to accommodate the forks of a standard forklift or a mobile self-loading packer (e.g., InnoLIFT 2200 lb capacity self-lifting loader), and the remainder of the roll-cage was designed to be taken apart. A horseshoe design was used for the docking mechanism with a self-guiding locking mechanism in order to help correctly position the scanner and pallet when loading.

For individual safety outside of the van, it is important that the 5 Gauss line perimeter be limited to the interior of the van. We verified this by measuring the magnetic field on the outside and around the van using a LATNEX MF-30 K Gauss Meter.

### Remote neuroimaging and data quality assessment

To demonstrate the ability to routinely collect at-home MRI data, MRI was performed with geometric phantoms and in vivo data collected from 12 individuals (6 female) from 4 to 40 years of age at their residence. ‘Reference’ in-lab scans were also collected from the same individuals on the same system but at our research lab to mimic the more conventional imaging center data collection. Images acquired at the residences and in-lab were visually inspected and compared for off-resonance and main field inhomogeneity artifacts, and mean length/width of the geometric phantom elements were calculated and compared. Signal-to-noise measures were also calculated and compared for mean frontal white matter and thalamic gray matter.

#### In vivo scanning

All in vivo human imaging was performed following informed consent and assent of the individual and parent or legal guardians under the direction of the host IRB at Rhode Island Hospital. All members of the research team had relevant Collaborative Institutional Training Initiative (CITI training), and first aid training. All methods were performed in accordance with the relevant guidelines and regulations. Informed consent and assent was also obtained to take, use, and publish photos of their homes, children, and self (e.g., Fig. 2) in print and online open-access publications.

#### Whole-brain T_2_-weighted fast spin echo anatomical scans were collected with the following parameters

TE/TR = 209/2000 ms; receiver bandwidth = 64 kHz, echo train length = 80; voxel resolution = 1.5 × 1.5 × 5 mm; and acquisition time of just under 6 min. To improve spatial resolution and image quality, the T2-weighted acquisition was repeated in the three orthogonal directions (axial, sagittal, and coronal), with super-resolution reconstruction^22^ performed to provide a final isotropic resolution 3D volume. Total acquisition time was approx. 17 min, including pre-scan calibration and localizer scans. While the Hyperfine Swoop is currently capable of T_1_, T_2_, and T_2_-FLAIR weighted anatomical imaging, we have restricted our analysis to T_2_-weighted data as we find it provides the best image contrast and quality^16^.

An atlas-based segmentation approach was used to delineate total white and gray matter, and cerebral spinal fluid. Here, each individual’s low-field data were first non-linearly aligned to age-corresponding anatomical templates in Montreal Neurological Institute (MNI) space^23^ using an automated three-dimensional registration approach (ANTS) with a mutual information (MI) cost function^24^. MI was used as opposed to the more common normalized cross-correlation metric to account for the contrast differences between the low-field images with T_2_-weighted contrast, and the higher resolution templates constructed from 3 T, T_1_-weighted MP-RAGE data. Using the inverse of this transformation, previously calculated high-resolution tissue masks were ‘reverse’ aligned to each individual’s low-field image. These registered masks were then used as priors for individual-level segmentations performed using the ANTS Atropos algorithm^25^. The Pearson correlation, and a paired t-test between the tissue volumes collected on the mobile and in-lab scans were then calculated and compared.

#### Geometric phantom

The same supplied standard Hyperfine geometric grid phantom was used to quantify potential geometric distortions on the mobile and in-lab scans. Using the same T_2_ acquisition approach for the in vivo scans, the phantom was scanned before each individual. Following acqusition, images were rotated in software to allow easier X and Y measurements and mean grid dimensions were calculated for each mobile and in-lab image pairs, and the Pearson correlated calculated and a paired t-test performed to identify potential geometric bias in the X and Y grid length. Subtle differences in slice position, owing to differences in the system-defined field-of-view, were not anticipated to affect the grid measures doe to the size and thickness of the phantom grid elements (i.e., > 2 × the image resolution).

## Conclusion

Accessible, lower-cost, and portable MRI systems offer the promise of mobile imaging based on a human-centered design philosophy in which the scanner and research lab comes to the participant. Here we have demonstrated, for the first time, a fully mobile MRI system that can reach almost any home in the US and offers quality whole-brain structural imaging without penalty to image quality of geometric fidelity. Results lay the foundation for larger-scale public health and epidemiological neuroimaging studies, potentially utilizing a network of connected mobile scanners, representing a fundamental shift from current standard approaches. While results here are shown in the US, we further envisage translating these results to lower income countries and settings, many of which have few or no MRI systems, with profound implications for global health and healthcare access.

## Data Availability

All data collected and presented in this publication are freely available through the corresponding author, SCD.
